# Rooting human parechovirus evolution in time

**DOI:** 10.1186/1471-2148-9-164

**Published:** 2009-07-15

**Authors:** Nuno R Faria, Michel de Vries, Formijn J van Hemert, Kimberley Benschop, Lia van der Hoek

**Affiliations:** 1 Department of Medical Microbiology, CINIMA, Academic Medical Center, University of Amsterdam, Amsterdam, The Netherlands

## Abstract

**Background:**

The *Picornaviridae *family contains a number of important pathogenic viruses, among which the recently reclassified human parechoviruses (HPeVs). These viruses are widespread and can be grouped in several types. Understanding the evolutionary history of HPeV could answer questions such as how long the circulating lineages last shared a common ancestor and how the evolution of this viral species is shaped by its population dynamics. Using both strict and relaxed clock Bayesian phylogenetics we investigated 1) the substitutions rates of the structural P1 and capsid VP1 regions and 2) evolutionary timescale of currently circulating HPeV lineages.

**Results:**

Our estimates reveal that human parechoviruses exhibit high substitution rates for both structural P1 and capsid VP1 regions, respectively 2.21 × 10^-3 ^(0.48 – 4.21 × 10^-3^) and 2.79 × 10^-3 ^(2.05 – 3.66 × 10^-3^) substitutions per site per year. These are within the range estimated for other picornaviruses. By employing a constant population size coalescent prior, the date of the most recent common ancestor was estimated to be at around 1600 (1427–1733). In addition, by looking at the frequency of synonymous and non-synonymous substitutions within the VP1 gene we show that purifying selection constitutes the dominating evolutionary force leading to strong amino acid conservation.

**Conclusion:**

In conclusion, our estimates provide a timescale for the evolution of HPeVs and suggest that genetic diversity of current circulating HPeV types has arisen about 400 years ago.

## Background

Parechoviruses belong to the *Picornaviridae *family which includes other pathogenic viruses such as foot-and-mouth disease virus (FMDV), hepatitis A virus, enteroviruses and rhinoviruses [1,2]. The *Parechovirus *genus includes two species: human parechoviruses (HPeV) and the zoonotic Ljungan virus. HPeV are non-enveloped pathogens with a single-stranded genomic RNA of positive polarity with around 7.400 nucleotides organized into a single long open reading frame in between a 5'UTR and 3'UTR. The open reading frame can be divided into three main regions: P1 (encoding capsid proteins VP0, VP3, VP1), P2 (nonstructural proteins) and P3 (nonstructural proteins, including the viral RNA polymerase) [2-4]. In HPeV, out of the three capsid proteins that constitute the monomeric units of the viral icosahedric-shaped capsid [3], VP1 protein plays a crucial role in cell entry via interaction of an Arg-Gly-Asp (RGD) triplet with integrins on the cell surface [5]. However, some HPeVs (among which the type 3 strains) lack the RGD motif in VP1, and their mode of cell recognition and entry is less clear [6]. Typing of HPeV is based on the VP1 sequence providing a reliable locus to type all the identified HPeV strains as described for enteroviruses by Oberste *et al *[7]. As a result, the majority of HPeV available nucleotide data concerns the VP1 gene.

In general, HPeV is transmitted by the oral-fecal route causing in most cases relatively mild respiratory and gastrointestinal symptoms [2,8], though conditions such as bronchiolitis [9] and severe neonatal infections [10,11] have also been reported. HPeV1 and HPeV2 were first isolated in 1956 and classified by serotyping as enteroviruses, respectively echovirus types 22 and 23 [3,12]. HPeV3 was first described in 2004 [6] and is associated with more severe conditions related to CNS symptoms [10,11,13]. Subsequently, improvements in HPeV-specific screening tools allowed a successful identification of HPeV4 and HPeV6 throughout North America, Japan and Europe [14-18]. Moreover, an HPeV variant originally classified as HPeV2-Connecticut was reclassified as HPeV5 [14]. Currently, sequences have become available for two novel types that were recently isolated in Pakistan and Brazil [19,20] and in the Netherlands one more novel type was identified (HPeV14, [21]). Unfortunately sequences of the HPeV types 9 to 13 are not available for analysis yet. Of all types, HPeV1 and HPeV3 are the most prevalent strains [11,22].

Understanding the mechanisms underlining pathogenicity and persistence of pathogens in human populations is an important aspect of disease epidemiology and control. Fixation of mutations into nucleotide substitutions, a key principle behind phylogenetic signatures, is shaped by major evolutionary forces such as selection (molecular adaptation deriving from an increasing fitness of a corresponding phenotypic trait) and genetic drift (stochastic gene sampling process at reproduction) [23,24]. A useful tool to detect and measure selection in viral gene sequences is the ratio between synonymous (dS) and non-synonymous (dN) substitutions. Whereas a ratio above 1.0 is an indicator of positive selection operating at the amino acid sequence level [25], significantly lower values are generally referred to as purifying selection and refer to preservation of the phenotypic trait.

RNA viruses yield the highest mutation rates of all groups of pathogens which is approximately six orders of magnitude higher than in most DNA organisms [23,26]. In the context of viral population genetics, substitution or evolutionary rates can be defined as the number of fixed mutational changes that accumulate in the population per nucleotide site per unit of time [27]. This rate is driven by the short-generation times of viruses and their error-prone RNA polymerase proteins lacking proofreading activity. Combined with their small genomes, these characteristics make RNA virus ideal models for evolutionary research [23,28,29]. In addition, recombination events may also play a role in RNA virus evolution [23]. While lacking a fossil record, evolutionary histories of RNA viruses can be calibrated because they represent 'measurably evolving populations', in which genetic diversity accumulates over a timescale of human observation [30]. Their evolutionary history and population dynamics can be reconstructed by means of genealogy-based coalescent approaches using nucleotide sequences sampled over an epidemiological time frame in order to estimate timed viral ancestry as well as the rates of genetic change [27,29]. The most advanced methods operating on time-stamped sequence data use Bayesian Metropolis-Hastings Markov Chain Monte-Carlo (MCMC) algorithms that accommodate for the uncertainty of phylogenies rooted in time. Here, we estimated the substitution rates for the P1 and VP1 regions of HPeV with such a Bayesian approach, which provides a statistical framework for evolutionary analysis [31].

The identification of several novel types within the last few years may be conceived as a relatively recent introduction of HPeV into the human population, but this is not necessarily the case. By reconstructing the evolutionary history of HPeV we shed light on this issue. We investigated when current HPeV diversity emerged by determining the time of divergence from the most recent common ancestor (TMRCA).

## Methods

### Sequence collection

Dataset 1 comprised 29 nucleotide sequences from the P1 structural region (2291 nt) from different HPeV isolates (12 sequences of HPeV1, 1 sequence of HPeV2, 4 sequences of HPeV3, 5 sequences of HPeV4, 2 sequences of HPeV5, 3 sequences of HPeV6, 1 sequence for HPeV7 and 1 sequence for HPeV8). Dataset 2 comprised 199 nucleotide sequences of the VP1 capsid region (647 nt) (117 sequences of HPeV1, 2 sequences of HPeV2, 40 sequences of HPeV3, 18 sequence of HPeV4, 9 sequence of HPeV5, 10 sequences of HPeV6, 1 sequence of HPeV7, 1 sequence of HPeV8 and 1 sequence of HPeV14). To date, sequences of HPeV9–13 have not been made available [32]. The accession numbers of the sequences from both data sets are available in Additional file 1. Sampling date (year) for dataset 1 (1956–2007) and for dataset 2 (1975–2007) was either collected directly from Genbank record or following direct contact with the relevant authors. Multiple alignments of the P1 and VP1 regions of HPeV were conducted in ClustalW [33] and sequences were edited manually with Se-Al v2.0 [34].

### Phylogenetic analysis

Overall evolutionary rates for P1 and VP1 regions were measured as the number of nucleotide substitution per site per year (s/s/y). Relevant parameters were summarized as the median of posterior distributions by Bayesian coalescent Markov chain Monte Carlo algorithm implement in the Bayesian Evolutionary Sampling Trees (BEAST) software package version 1.4.8 [31]. To identify the optimal substitution model we performed a maximum likelihood analysis using the Modelgenerator package [35]. The model that best fit both sequence datasets was General Time Reversible (GTR) model with a discretised γ-distribution (GTR+Γ), allowing for nucleotide rates to vary among sites within the protein coding sequence alignments. Codon partitions (1+2)+3 were applied to both alignments, keeping first and second positions (mostly to non-synonymous changes) in one partition and the third position (related to increase redundancy and prone to synonymous changes) in a separate partition [36]. Relative rate parameters were estimated in separate for each partition, in order to accommodate rate variation at the third codon position.

We employed both strict and relaxed lognormal molecular clocks, the latter allowing rate variation among branches [37]. The coefficient of variation (σ_r_) was used as a quantification of the rate variation among branches (σ_r _> 0.2 was considered as significant rate variation among branches) (Table 1). A constant size demographic model was used as coalescent prior. Each alignment of both data sets was analyzed using Markov Chain Monte-Carlo (MCMC) computations run over a sufficient time to achieve convergence of the chains, which was analyzed by inspection of the MCMC samples using TRACER 1.4 [38]. The 95% highest posterior density (HPD) interval is the shortest credible interval that contains 95% of the samples values. Statistical uncertainties of the substitution rates and the TMRCA were summarized as the lower 95%, median, and upper 95% values of the HPD. Out of the tested models (GTR + Γ, both with strict and relaxed lognormal molecular clocks), the clock model that performed better was the lognormal molecular clock, which yielded the highest marginal likelihood. Clock models were also compared in terms of Bayes Factors (BF, Table 2). The relaxed model clock following a lognormal distribution was also supported by the highest log_10 _BF as suggested [39].

**Table 1:** Statistical parameters estimates from BEAST analysis under a strict and relaxed molecular clock

**Genetic region**	**Molecular clock**	**Coefficient of variation (σ_r_)**	**Prior** **Probability^a^**	**Marginal** **Posterior^a^**
P1	Strict	-	-183.8 (199.6, 167.4)	-23989 (24007, 23971)
	Lognormal	0.29	-180 (154, 216)	-23968 (24003, 23934)

VP1	Strict	-	-1407 (1457, 1350)	-19148 (19208, 19094)
	Lognormal	0.41	-1362 (1433, 1301)	-19033 (19103, 18964)

^a^Mean prior and marginal posterior probabilities for both clock models are given with 95% lower and upper bounds of the highest probability density intervals in parentheses. The coefficient of variation is a measure of the rate variation among branches (see Methods for details).

**Table 2:** HPeV P1 and VP1 substitution rates and TMRCA estimates under a strict and relaxed lognormal molecular clock

**Genetic region**	**Molecular clock**	**Substitution rate** **(× 10^-3^)^a^**	**TMRCA^b^**	**Log_10 _BF^c^**
P1 gene	Strict	2.03 (1.15, 2.91)	1581 (1334 – 1733)	-7.03 ± 0.3
	Lognormal	**2.21 (0.48, 4.21)**	**1603 (940 – 1883)**	0.0

VP1 gene	Strict	2.30 (1.74, 2.90)	1553 (1412 – 1673)	-27.8 ± 0.2
	Lognormal	**2.79 (2.05, 3.66)**	**1600 (1427 – 1733)**	0.0

^a ^The median estimates of the substitution rates and ^b^TMRCA for the P1 and VP1 region are shown for each molecular clock with 95% lower and upper bounds of the highest probability density intervals in parentheses. ^c ^The log_10 _Bayes factor (BF) difference (± standard error) in estimated harmonic median of the marginal likelihoods (with the posterior as distribution probability) compared with the clock model with strongest support. For each dataset the highest -log10 Bayes factor corresponds to the best-fit clock model (highlighted in bold).

The fact that a relaxed lognormal molecular clock fits best to our data was consistent with an estimated coefficient of variation of 0.29 and 0.41 (respectively, for dataset comprising P1 and VP1 regions) that reflected significant rate heterogeneity, thus rejecting a strict molecular clock. The resulting trees for each run were summarized using TreeAnnotator and the maximum clade credibility tree was visualized with FigTree v1.1.2 [34]. BEAST xml files are available as additional files 2, 3, 4 and 5.

### Detection of adaptative molecular evolution

Overall selective pressures acting on VP1 antigenic region were estimated by using the CODEML program in the PAML package [40]. We used site models M7 (with a discrete distribution of 10 categories and accounting for sites not allowed to be positively selected) and M8 (estimates dN/dS for an extra class (p11) of sites, accounting for positively selected sites with dN/dS>1). Models were compared by means of likelihood ratio test and statistical support was taken from the Bayes-Empirical-Bayes output (BEB, see additional file 6: Log-likelihood and parameter estimates for PAML analysis) [40]. To detect adaptative molecular evolution, we used the complete dataset 2.

## Results and discussion

### Rate of evolutionary change for P1 and VP1 regions of HPeV

We first identified the best-fitting substitution model for the HPeV sequences using the Modelgenerator package (GTR + Γ) [35], and tested whether the evolution of the P1 and VP1 genetic regions was better described by a strict or relaxed lognormal molecular clock. A relaxed lognormal molecular clock provided a better fit to both datasets according to Bayes Factor (BF) analyses (P1: log_10 _BF = 7.03 and VP1: log_10_BF = 27.8, Table 2). This is in accordance with significant rate variation among the branches of the inferred phylogeny as measured by a non-zero coefficient of variation (σ_r_) obtained with the relaxed molecular clock analysis (P1: σ_r _= 0.29; VP1: σ_r _= 0.41) (see Methods for details). Using the available P1 and VP1 dated sequences of HPeV, our analysis inferred a similar rate of nucleotide substitution for both regions (P1 median: 2.21 × 10^-3 ^s/s/y, 95% HPD [0.48 × 10^-3^, 4.21 × 10^-3^]; VP1 median: 2.79 × 10^-3 ^s/s/y, 95% HPD [2.05 × 10^-3^, 3.66 × 10^-3^]) (Table 2). The higher rate indicated for the VP1 region is possibly related to its antigenic properties, perhaps reflecting a difference in the level of gene expression or mirroring the involvement of the VP1 capsid protein in the viral entry mediated by cellular integrins.

Despite our study focused on the available sequences of HPeV, more accurate estimates could probably be obtained with broader and more homogenous sampling timescale, preferably for all types. Yet, this may be a daunting task because it is difficult to obtain older samples and some of the HPeV types e.g. HPeV2, HPeV4, HPeV5 and HPeV6 appear to be relatively rare (see e.g[17,18]). Moreover, a common pitfall on estimating evolutionary rates is its underestimation due to mutational saturation of synonymous sites [41-44]. By using a gamma distributed substitution model, we assured that rate variation among sites was allowed. Therefore, the effect of possible saturation of synonymous sites was alleviated by permitting a proportion of these sites to change at a higher rate [43]. In addition, we used partitioning in codon positions that allows different codon positions to have different substitution rates (and different amount of rate heterogeneity) (see Methods for details) [31,45] thus further accommodating rate variation among synonymous and non-synonymous positions.

The high rates of evolutionary change obtained in this study are in accordance with the evolutionary rates of other RNA viruses [32,33]. Consistently, HPeV replication mechanism relies on an RNA-dependent RNA polymerase that lacks proofreading capacity. This increases the number of mutations incorporated in viral genomes over time and settles the ground for a relatively rapid genetic diversification [46]. The evolutionary rates of a few members of the *Picornaviridae *family have been studied. Despite the fact that most of the studies used different evolutionary frameworks, the rate of evolutionary change estimated in this study for the capsid region of HPeV VP1 is 1) faster than the rate of Hepatitis A virus [47], 2) resembles the rate estimated for the antigenic region of Echovirus 71 [41,48,49] and finally 3) it is nearly one order of magnitude lower than the rates of poliovirus (2.09 × 10^-2 ^s/s/y) [48] or FMDV (2.7 × 10^-2 ^s/s/y) [50].

RNA viruses are the most suitable object of study for rates of change and divergence times. This is due in large part to the rapid rate at which they evolve allowing genetic diversity to accumulate within a timescale approximately the same as mutations are fixed in viral populations [29]. Yet, a deeper understanding of the replication machinery of HPeV (e.g. generation times, fidelity of RNA polymerase) may deliver insights on the molecular basis of these high rates of evolutionary change [44].

### Timescale of HPeV evolution

**Figure 1:**
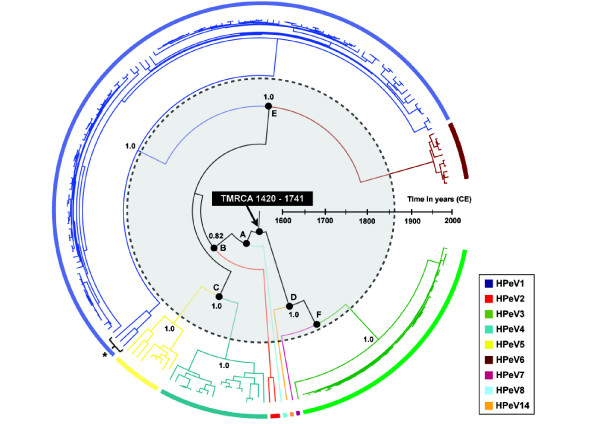
**Bayesian time-scaled phylogeny of HPeV based on VP1 sequence analysis**. Maximum clade credibility tree obtained with BEAST with a constant size coalescent prior showing lineage splitting events (nodes A-F) since the most recent common ancestor to the presently circulating HPeV types. The divergence times correspond to the mean posterior estimate of their ages (in years). For the TMRCA, the correspondent 95% Bayesian credible intervals are shown (median 1600). Time axis is shown in years and ranges from the TMRCA to the present year. Deeper and some subtype nodes with posterior probability of higher than 0.8 are pointed out. Each colour corresponds to a specific HPeV, as indicated in the box on the right. The dashed grey circle depicts the extent of genetic diversity of the sampled HPeV strains. HPeV-1-"Harris-like" strains (*) clustered separately from the contemporary HPeV-1.

According to our analysis based on 199 HPeV VP1 available sequences, these viral species diverged from their most recent common ancestor (MRCA) at the year 1600 (95% HPD [1427–1733]) (Figure 1, Table 2). Moreover, and focusing on the two most recently isolated types (HPeV7 [19], HPeV8 [20]) our findings indicate that HPeV7 diverged from HPeV3 around 150 years ago (1854, 95% HPD [1747–1936] (Figure 1, F) and HPeV8 seems to have diverged from the group of HPeV3, HPeV7 and HPeV14 at around and 315 years ago (1726, 95% HPD [1519–1855]). HPeV14 (isolate 451564, accession number FJ373179) is still under completion and thus far only its VP1 sequence is available for analysis (Benschop *et al*. personal communication and [21]). However, divergence of HPeV14 (from HPeV7 and HPeV3) could be estimated at around 220 years ago (1788, 95% HPD [1636–1902]). Taken together, we suggest that the genetic diversity of the currently circulating HPeV types has arisen around 400 years ago (Figure 1).

The wider 95% Bayesian credible intervals obtained for the estimates using dataset 1 composed by the total of 29 available P1 sequences to date (Table 2) probably reflect a less heterochronous sequence data. Yet, an identical timescale was obtained when performing the MCMC approach with the dataset comprising the P1 region (1603, 95% HPD [940–1883]) (Table 2). Despite holding new pieces to solve the puzzle of HPeV origins, the evolutionary rates and the timescales for the most recent common ancestor and type lineage-splitting events, may be better framed once a larger number of sequences are available [51]. However, the overlapping of the 95% Bayesian credible intervals obtained in our analysis for both genomic regions indicates that our estimates on the TMCRA of the HPeV lineages are robust (Table 2).

One facet of fast evolving RNA viruses that induce acute infections (as the case of HPeV) is that they are likely candidates for jumps between species boundaries [29]. While the latter appears to be clearly established for e.g. SARS-CoV or influenza H5N1, a zoonotic link remains to be elucidated for HPeV. Because Ljungan virus shares a close phylogenetic proximity with HPeV virus, it is likely that both species have had a common ancestor [52]. Moreover, the reservoir host for Ljungan virus is *Myodes glareolus*, a widely distributed rodent commonly named as bank vole [4]. Despite the connection of Ljungan virus infection and human disease still remains to be clarified, bank voles are recognized as the reservoirs of other infectious agents, e.g. Puumala Hantavirus [53] and have been linked to a significant number of outbreaks over Europe [54-56].

### Purifying selection is dominant in HPeV evolution

**Figure 2:**
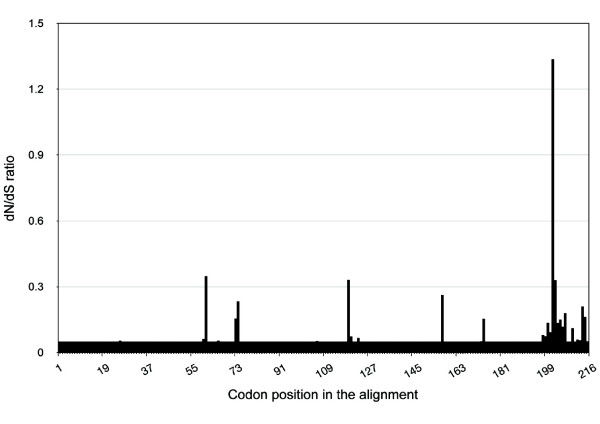
**The dN/dS ratios per site in VP1 region of HPeV**. Rate of nonsynonymous-to-synonymous substitutions per codon site across the VP1 region of the HPeV genome. The only amino acid likely prone to molecular adaptation (dN/dS > 1.0) at position 202 does not have sufficient statistical support (see also additional file 7).

In search for the driving force that shapes the evolution of the HPeVs, we looked at the ratio of non-synonymous-to-synonymous substitutions (the dN/dS ratio) [24]. For most codons in the VP1 region the ratio is < 0.1 (Figure 2). We noticed a few sites that tend to escape from purifying selection displaying dN/dS values > 0.3 (position Q61, A119, G203), or even > 1.0 (position N202 of our alignment, see additional file 7), however with statistically poor support (see additional file 6, Log-likelihood and parameter estimates for PAML analysis).

Also other studies have found an overall low dN/dS ratio for the HPeVs [1,57]. Our analysis confirms on a codon level that throughout the structural region strong purifying selection is dominant, leading to the conservation at the level of the amino acid sequence. Future analysis may shed lights not only in a unified framework of evolution for this viral species but also help preventing major burdens associated with HPeV pathogenicity.

## Conclusion

The HPeV are highly prevalent human RNA viruses and thus far no studies have addressed the evolutionary history of these pathogens. The Bayesian analysis presented here first indicates that the structural P1 and the capsid VP1 region of this viral species evolve at a high rate of evolutionary change (~10^-3 ^substitutions per site per year). Additional genomic and epidemiological data will help to reveal the relation between such rates and the widespread of this viral species. We also show that the currently circulating HPeV types have shared a common ancestor around four centuries ago. Since then, HPeV evolved into different lineages that have spread widely. Overall, a strong tendency for phenotypic conservation could be observed, suggesting that genetic drift plays an important role in the generation of the diversity within the regions under investigation. In summary, by delivering insights into the evolutionary mechanisms of HPeV, this study provides the foundations for a unified understanding of HPeV evolution.

## Authors' contributions

NRF and LvdH designed and conceived the study. MV, NRF and KB collected and aligned the sequences. NRF and FJH carried out the analysis and analyzed the data. NRF and LvdH wrote the paper. All authors read and approved the final manuscript.

## Supplementary Material

Additional file 1 **Genbank accession numbers of the sequences**. The file provides the accession number of HPeV P1 (dataset 1) and VP1 (dataset 2) sequences, including respective isolate names and sampling dates. Click here for file

Additional file 2 **BEAST XML file for the HPeV-P1-relaxed analysis**. Input XML file used for BEAST relaxed molecular clock analysis of P1 region (dataset 1). Click here for file

Additional file 3 **BEAST XML file for the HPeV-P1-strict analysis**. Input XML file used for BEAST strict molecular clock analysis of P1 region (dataset 1). Click here for file

Additional file 4 **BEAST XML file for the HPeV-VP1-relaxed analysis**. Input XML file used for BEAST relaxed molecular clock analysis of VP1 region (dataset 2). Click here for file

Additional file 5 **BEAST XML file for the HPeV-VP1-strict analysis**. Input XML file used for BEAST strict molecular clock analysis of VP1 region (dataset 2). Click here for file

Additional file 6 **Log-likelihood and parameter estimates for PAML analysis**. The data provided represent the log-likelihood and Bayes-Empirical-Bayes output (models 7 and 8) for estimating dN/dS ratios of the HPeV VP1 region (dataset 2). Click here for file

Additional file 7 **PAML VP1 codon alignment**. Zipped NEX file. Codon alignment of HPeV VP1 region used for PAML estimates. Click here for file
