# Supplementary material for: Rooting human parechovirus evolution in time
Source: BMC Evol Biol. 2009 Jul 15;9:164. doi: 10.1186/1471-2148-9-164 (PMC2723090; doi:10.1186/1471-2148-9-164)
Supplement: Additional file 1 — Genbank accession numbers of the sequences. The file provides the accession number of HPeV P1 (dataset 1) and VP1 (dataset 2) sequences, including respective isolate names and sampling dates. [file 1471-2148-9-164-S1.pdf]

Dataset 1 (P1 region)

Accession number	HPeV type	Isolate	Sampling time
S45208	1	Harris	1956
AB112485	1	A1086-99	1999
AB112487	1	A10987-00	2000
FM178558	1	7555312	2002
EU024636	1	BNI-R32/03	2003
EU024635	1	BNI-R30/03	2003
EU024634	1	BNI-R21/03	2003
EU024633	1	BNI-R15/03	2003
EU024632	1	BNI-R09/03	2003
EU024630	1	BNI-90	2003
EF051629	1	BNI-788st	2004
NC_001897	2	Gregory	1956
AB112483	3	A354-99	1999
AB112482	3	A317-99	1999
AB084913	3	A308-99	1999
AJ889918	3	Can82853-01	2001
AM235750	4	T75-4077	1979
AB434673	4	NII370-93	1993
AB433630	4	Fuk2001-282	2001
DQ315670	4	K251176-02	2002
AB433629	4	Fuk2005-123	2005
AF055846	5	CT86-6760	1986
AM235749	5	T92-15	1992
EU077511	5	2000-1108-2	2000
AB252582	6	NII561-2000	2000
EU024629	6	BNI-67/03	2003
EU077518	6	2005-823	2005
EU556224	7	PAK5045	2007
EU716175	8	BR/217/2006	2006

Dataset 2 (VP1 region)

Accession number	HPeV Type	Isolate	Sampling year
EU077503	1	1999-1096	1999
AM933170	1	69960AE	2000
AM933171	1	70094A	2000
DQ172438	1	152598	2001
DQ172439	1	252581	2002
DQ172434	1	252521	2002
DQ172433	1	252228	2002
FM178558	1	7555312	2002
DQ172437	1	350757	2003
EU024636	1	BNI-R32	2003
EU024635	1	BNI-R30	2003
EU024634	1	BNI-21	2003
EU024633	1	BNIR15	2003
EU024632	1	BNI-R09/03	2003
EU024631	1	BNI-R04	2003
EU024630	1	BNI-90/03	2003

DQ172435	1	452252	2004
DQ172441	1	452538	2004
DQ172440	1	451294	2004
DQ172436	1	452712	2004
EU077515	1	2004-1608	2004
FJ373083	1	452786	2004
FJ373082	1	452666	2004
FJ373081	1	452643	2004
FJ373080	1	452568	2004
FJ373079	1	452530	2004
FJ373078	1	452476	2004
FJ373077	1	452344	2004
FJ373076	1	452208	2004
FJ373075	1	451894	2004
FJ373074	1	451858	2004
FJ373073	1	451821	2004
FJ373072	1	451729	2004
FJ373071	1	451669	2004
FJ373070	1	451660	2004
FJ373069	1	451302	2004
FJ373068	1	451125	2004
FJ373067	1	450829	2004
FJ373066	1	450823	2004
FJ373065	1	450734	2004
FJ373064	1	450653	2004
FJ373063	1	450559	2004
FJ373062	1	450342	2004
FJ373061	1	450308	2004
FJ373060	1	450296	2004
FJ373059	1	450111	2004
EF051629	1	BNI-788st	2004
DQ172416	1	54330	2005
EU077520	1	2005-939	2005
EU077519	1	2005-938	2005
FJ373101	1	552762	2005
FJ373100	1	552444	2005
FJ373099	1	552314	2005
FJ373098	1	552154	2005
FJ373097	1	552001	2005
FJ373096	1	551907	2005
FJ373095	1	551612	2005
FJ373094	1	550981	2005
FJ373093	1	550652	2005
FJ373092	1	550505	2005
FJ373091	1	550435	2005
FJ373090	1	550332	2005
FJ373089	1	550328	2005
FJ373088	1	550320	2005
FJ373087	1	550290	2005
FJ373086	1	550252	2005
FJ373085	1	550193	2005
FJ373084	1	550182	2005
FJ373136	1	677033	2006

FJ373135	1	677008	2006
FJ373134	1	676406	2006
FJ373133	1	676323	2006
FJ373132	1	676271	2006
FJ373131	1	675458	2006
FJ373130	1	674450	2006
FJ373129	1	653096	2006
FJ373128	1	652920	2006
FJ373127	1	652780	2006
FJ373126	1	652643	2006
FJ373125	1	652568	2006
FJ373124	1	652536	2006
FJ373123	1	652499	2006
FJ373122	1	652467	2006
FJ373121	1	652445	2006
FJ373120	1	652281	2006
FJ373119	1	652146	2006
FJ373118	1	651934	2006
FJ373117	1	651898	2006
FJ373116	1	651654	2006
FJ373115	1	651625	2006
FJ373114	1	651128	2006
FJ373113	1	651108	2006
FJ373112	1	650989	2006
FJ373111	1	650941	2006
FJ373110	1	650854	2006
FJ373109	1	650750	2006
FJ373108	1	650648	2006
FJ373107	1	650606	2006
FJ373106	1	650258	2006
FJ373105	1	650164	2006
FJ373104	1	650163	2006
FJ373103	1	650151	2006
FJ373102	1	650081	2006
FJ648741	1	JP-8282	2007
FJ648754	1	JP-8311	2007
FJ648753	1	JP-8457	2007
FJ648752	1	JP-8430	2007
FJ648751	1	JP-8312	2007
FJ648750	1	JP-8304	2007
FJ648749	1	JP-8277	2007
FJ648748	1	JP-8262	2007
FJ648747	1	JP-8462	2007
FJ648746	1	JP-8329	2007
FJ648745	1	JP-8323	2007
FJ648744	1	JP-8276	2007
FJ648743	1	JP-8243	2007
FJ648742	1	JP-8150	2007
FJ648760	2	T144	2005
EU360514	2	NO-13631	2006
EU077509	3	2000-976	2000
EU077507	3	2000-759	2000
EU077506	3	2000-752	2000

EU077504	3	2000-632	2000
DQ172442	3	152037	2001
DQ172451	3	252277	2002
DQ172450	3	250956	2002
DQ172448	3	251393	2002
DQ172445	3	251407	2002
DQ172444	3	251360	2002
DQ172443	3	251181	2002
DQ172449	3	451371	2004
DQ172447	3	451517	2004
DQ172446	3	450936	2004
FJ373152	3	452000	2004
FJ373151	3	451987	2004
FJ373150	3	451935	2004
FJ373149	3	451692	2004
FJ373148	3	451678	2004
FJ373147	3	451677	2004
FJ373146	3	451653	2004
FJ373145	3	451610	2004
FJ373144	3	451550	2004
FJ373143	3	451513	2004
FJ373142	3	451512	2004
FJ373141	3	451425	2004
FJ373140	3	451393	2004
FJ373139	3	451377	2004
FJ373138	3	451297	2004
FJ373137	3	450900	2004
FJ373162	3	677146	2006
FJ373161	3	676401	2006
FJ373160	3	676053	2006
FJ373159	3	675391	2006
FJ373158	3	674271	2006
FJ373157	3	652880	2006
FJ373156	3	652761	2006
FJ373155	3	652649	2006
FJ373154	3	652545	2006
FJ373153	3	651689	2006
AM234725	4	T73-838	1973
AM235750	4	T75-4077	1975
AM933169	4	T73-510	1975
AM933168	4	T75-4080	1979
AM234727	4	T82-203	1982
AB434673	4	NII370-93	1993
AB433630	4	Fuk2001-282	2001
DQ315670	4	K251176_02	2002
FJ373166	4	452674	2004
FJ373165	4	452524	2004
FJ373164	4	452323	2004
FJ373163	4	450369	2004
EU077521	4	2006-99	2006
FJ373171	4	675149	2006
FJ373170	4	653046	2006
FJ373169	4	652872	2006

FJ373168	4	652598	2006
FJ373167	4	652580	2006
AM234728	5	T82-0169	1982
AM234726	5	T82-659	1982
AM234724	5	T83-2051	1983
AM933167	5	T83-456	1983
AM235749	5	T92-15	1992
FJ373172	5	452373	2004
FJ373175	5	676618	2005
FJ373173	5	552106	2005
FJ373174	5	652444	2006
AB252582	6	NII561-2000	2000
EU024629	6	BNI-67	2003
FJ373176	6	451701	2004
EU077518	6	2005-823	2005
FJ373177	6	550389	2005
EU360556	6	NO-7923	2005
EU360541	6	NO-7362	2005
EU360532	6	NO-4662	2005
EU360527	6	NO-4324	2005
FJ373178	6	650045	2006
EU556224	7	PAK5045	2007
EU716175	8	BR/217/2006	2006
FJ373179	14	451564	2004
