# Supplementary material for: Rooting human parechovirus evolution in time
Source: BMC Evol Biol. 2009 Jul 15;9:164. doi: 10.1186/1471-2148-9-164 (PMC2723090; doi:10.1186/1471-2148-9-164)
Supplement: Additional file 6 — Log-likelihood and parameter estimates for PAML analysis. The data provided represent the log-likelihood and Bayes-Empirical-Bayes output (models 7 and 8) for estimating dN/dS ratios of the HPeV VP1 region (dataset 2). [file 1471-2148-9-164-S6.doc]

## Additional file 6 - Log-likelihood and parameter estimates for PAML analysis

PAML CODEML models applied for the determination of dN/dS ratios at codon sites. The transition/transversion ratio is 5.7. The log likelihood values indicate a preference for model8 compared to model7. Values for p and q indicate the dN/dS ratio as a function of the proportion of sites with a certain dN/dS ratio, i.e., the parameters for the β-distribution. The p11 class of model8 contains the sites with dN/dS ratios >1.

| Model | Parameter | |
| --- | --- | --- |
| 7 | ts/tv | 5.710 |
|  | lnL | -19093.834934 |
|  | *p* | 0.25949 |
|  | *q* | 5.77255 |
| 8 | ts/tv | 5.715 |
|  | lnL | -19093.097831 |
|  | *p* | 0.26168 |
|  | *q* | 6.04305 |
|  | p0 | 0.99627 |
|  | dN/dS (p11) | 1.52983 (0.00373) |
